# Identification of Biomarkers for Lung Adenocarcinoma With Qi Deficiency and Phlegm Dampness

**DOI:** 10.1111/crj.13812

**Published:** 2024-08-06

**Authors:** Jiabin Chen, Sheng Wang, Qiaolei Yang, Yongjun Zhang, Jianfei Shen, Kequn Chai

**Affiliations:** ^1^ Department of Oncology Tongde Hospital of Zhejiang, affiliated to Zhejiang Chinese Medicine University Hangzhou China; ^2^ Department of Respiratory Jinhua Guangfu Hospital Jinhua China; ^3^ Institute of Pharmaceutical Biotechnology, Faculty of Medicine Zhejiang University Hangzhou China; ^4^ Department of Integrated Chinese and Western Medicine Cancer Hospital of University of Chinese Academy of Sciences Hangzhou China; ^5^ Department of Thoracic Surgery Taizhou Hospital Taizhou China

**Keywords:** biomarker, lung adenocarcinoma, Qi deficiency and phlegm dampness, RNA‐seq, traditional Chinese medicine

## Abstract

**Background:**

Qi deficiency and phlegm dampness (QPD) is one of the most common traditional Chinese medicine (TCM) syndromes in lung adenocarcinoma (LUAD). This study aimed to identify syndrome‐specific biomarkers for LUAD with QPD syndrome.

**Methods:**

Peripheral blood mononuclear cells (PBMCs) from LUAD patients with QPD, LUAD patients with non‐QPD (N‐QPD), and healthy control (H) were collected and analyzed with RNA‐seq to identify differentially expressed genes (DEGs). The area under the receiver operator characteristic curve (AUC) of each DEG was calculated, and the top 10 highest AUC DEGs were validated by qRT‐PCR. Logistic regression analysis was used to develop a diagnostic model evaluated with AUC.

**Results:**

A total of 135 individuals were enrolled in this study (training set: 15 QPD, 15 N‐QPD, 15 H; validation set: 30 QPD, 30 N‐QPD, 30 H). A total of 1480 DEGs were identified between QPD and N‐QPD. The qRT‐PCR results showed that the expression of DDR2 was downregulated, and PPARG was upregulated, which was in line with the finding of the training set. We developed a diagnostic model with these two genes. The AUC of the diagnostic model in the training cohort and validation cohort was 0.891 and 0.777, respectively.

**Conclusions:**

We identified the two genes (DDR2 and PPARG) as syndrome‐specific biomarkers for LUAD with QPD syndrome and developed a novel diagnostic model, which may help to improve the accuracy and sensibility of clinical diagnosis and provide a new target for natural drug treatment of LUAD.

## Graphical abstract

The DDR2 and PPARG were identified as potential biomarkers for LUAD with QPD syndrome. The practical diagnostic model based on DDR2 and PPARG enriched the diagnostic methods of TCM syndrome differentiation, which may help to improve the accuracy and sensibility of clinical diagnosis. The practical diagnostic model may provide a new target for natural drug treatment of LUAD.

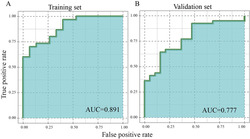

## 1 Introduction

Lung cancer (LC) remains the most common cancer and the leading cause of cancer‐related deaths globally [1]. It was estimated that more than 234 000 new cases were diagnosed as LC, and over 170 000 patients die of LC annually [2, 3]. Lung adenocarcinoma (LUAD) is the primary pathological subtype of non‐small cell LC (NSCLC), accounting for 50% of NSCLC. The standard therapies for LUAD are surgical resection, chemotherapy, and radiotherapy, and they have significantly improved the clinical outcomes of LUAD [4]. However, when LUAD patients are treated with chemotherapy and radiotherapy, considerable side effects often occur, such as nausea and vomiting, decreases in white blood cells, and gastrointestinal reactions [5].

Traditional Chinese medicine (TCM) has been used to treat and prevent cancer for over 2000 years. It has shown promising efficacy in cancer adjuvant therapy by improving the quality of life, reducing the side effects of radiotherapy and chemotherapy, and prolonging survival time [6, 7]. Syndrome differentiation is the core theory of TCM, indicating that complementary treatment should be adopted according to different types of TCM syndromes [8]. However, it is challenging to discriminate TCM symptoms only by clinical symptoms, and the lack of laboratory diagnosis and specific clinical biomarkers increased the difficulty. Qi deficiency and phlegm‐turbid stagnation are one of the most common and typical TCM syndromes in LC (15.8–21.9%) [9]. Qi deficiency and phlegm dampness (QPD) constitution are mostly caused by the weakness of the spleen and stomach [10]. The main symptoms of Qi deficiency are physical weakness, dizziness, fatigue, and pulse deficiency. The main symptoms of phlegm dampness are easy sweating, greasy skin, phlegm, and chest tightness. We formulated systematically the theoretical point of view that treats malignant tumors from eliminating phlegm to detoxification and have achieved survival benefits in LC treatment under theoretical guidance [11, 12]. Moreover, a previous study has reported that therapeutic methods of eliminating phlegm and others play compound functions in cancer patients and formulate an exemplary method for alleviating the side effects of both radiotherapy and chemotherapy [13]. Meanwhile, the prescription of eliminating phlegm has been identified as inhibiting the growth and metastasis of Lewis lung carcinoma in mice remarkably [14]. TCM syndromes rely on the subjective judgment of experienced TCM practitioners and lack an accurate diagnostic strategy. The combination of TCM and modern molecular medicine will contribute to the accurate diagnosis of TCM symptoms.

**FIGURE 1:**
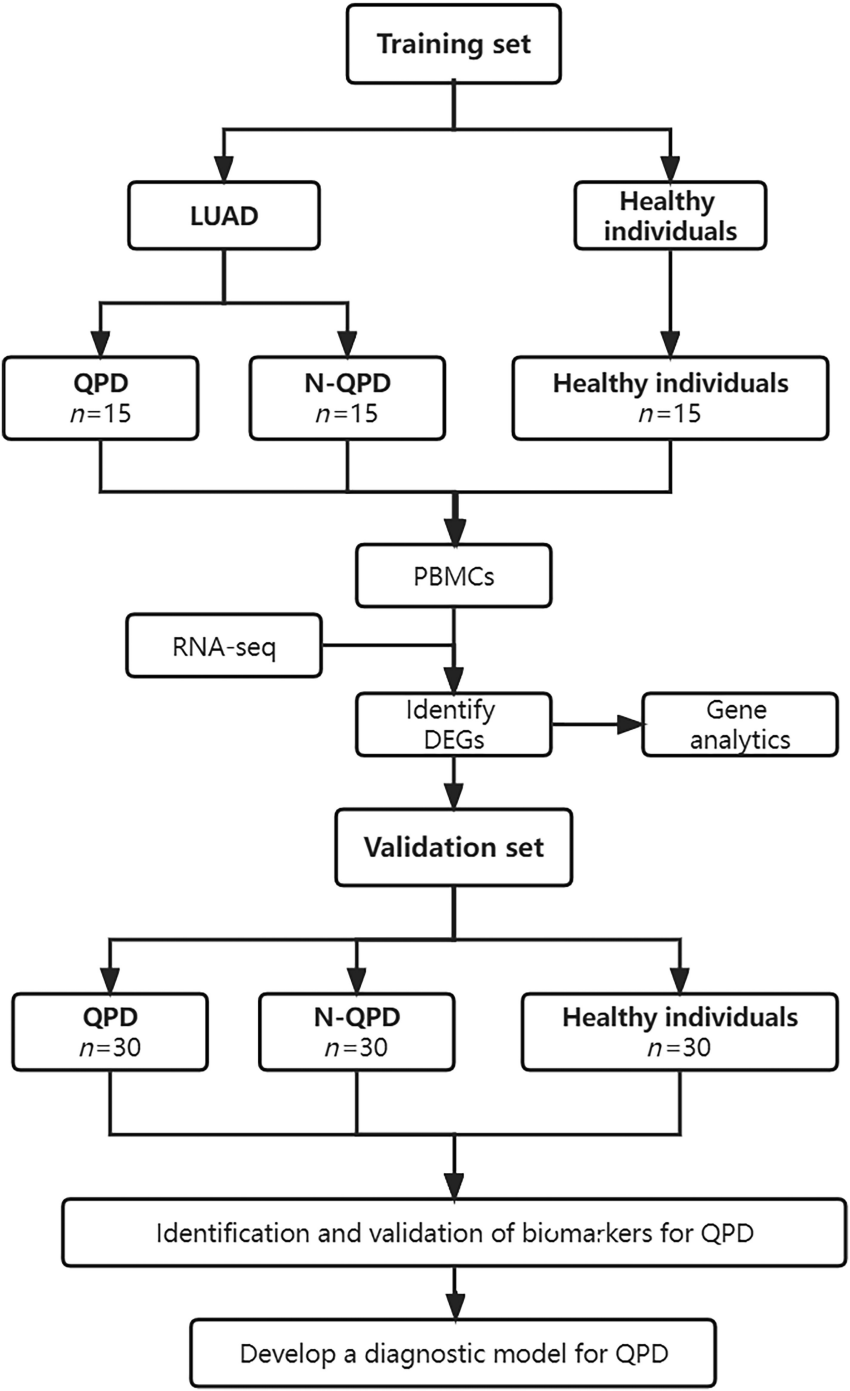
The detailed study flow in the study. LUAD: lung adenocarcinoma; QPD: Qi deficiency and phlegm dampness; N‐QPD: non‐QPD; PBMCs: peripheral blood mononuclear cells.

Recently, omics technology has provided a novel way to systematically understand the potential mechanism of cancers at the molecular level and could be utilized for identifying novel biomarkers for cancer treatment and diagnosis. In the present study, we collected peripheral blood mononuclear cells (PBMCs) from LUAD patients with QPD, LUAD patients with non‐QPD (N‐QPD), and healthy individuals (H). We analyze them with RNA‐seq to identify syndrome‐specific biomarkers for LUAD with QPD. Then, qRT‐PCR was used for validating syndrome‐specific biomarkers, and logistics regression analysis was used to develop a diagnostic model, which may help provide evidence‐based medical support for developing TCM theory. A detailed flow chart of this study is shown in Figure 1.

## 2 Materials and Methods

### 2.1 Criteria for LUAD and TCM Syndrome

Between July 2016 and June 2018, a total of 45 subjects (training cohort: 15 QPD, 15 NQPD, 15 H; validation cohort: 30 QPD, 30 NQPD, 30 H) were collected from three independent research centers (Tongde Hospital, Cancer Hospital of University of Chinese Academy of Sciences, and Taizhou Hospital).

Patients meeting the following criteria were included: (1) patients aged > 18 years and < 75 years with histologically/cytologically confirmed LUAD; (2) life expectancy > 6 months; (3) patients without surgical operation, chemotherapy, radiotherapy, biological therapy, and TCM treatment previously; (4) patients with Karnofsky (KPS) ≥ 60 and Eastern Cooperative Oncology Group (ECOG) ≤ 2.

Patients meeting one or more of the following criteria were excluded: (1) patients with any severe acute and chronic medical condition; (2) pregnant or breastfeeding women; (3) abnormal liver function (hemobilirubin > 1.5 ULN [upper limit of average value], ALT, AST > 2.5 ULN), kidney function (serum creatinine > 1.5 ULN), and blood picture (hemoglobin < 100 g/L, white blood cell < 4 × 10^9^/L, neutrophils < 2 × 10^9^/L, platelet < 100 g/L).

The TCM syndrome was judged with characteristics according to the Chinese Medical Constitution 2008, Issued by the Chinese Association of TCM and TCM Clinical Diagnosis and Treatment Terminology. Meanwhile, the results were confirmed by three Professors of TCM. This study was approved with informed consent by the ethics committee of Tongde Hospital, Taizhou Hospital, and Cancer Hospital.

### 2.2 Sample Collection and PBMC Isolation

After overnight fasting, 10 mL of peripheral blood was collected in an EDTA tube and stored at −80°C. Before analysis, the peripheral blood samples were thawed to room temperature. An aliquot of 300 μL plasma samples was placed in 1.5 mL Eppendorf tubes and added 900 μL FACS Lysing Solution. After 10 min, each sample was centrifuged at 3000 rpm for 15 min. Then, the supernatant was removed, and the residue was resuspended in a solution (90% FBS and 10% DMSO) and stored in liquid nitrogen.

### 2.3 RNA‐seq Analysis

Qubit 2.0 (Life Technologies, USA) and Bioanalyzer 2100 (Agilent, Germany) were used to analyze the quality and integrity of RNA. For library preparation, 3 μg of total RNA were captured by NEBNext Oligos (T) 25 beads (NEB, USA), sheared to yield fragments of ~250 bp, and reverse‐transcribed with NEBNext RNA first and second Strand Synthesis Module (NEB, USA). The products were end‐repaired, A‐tailed, ligated to Illumina sequencing adapters, and amplified by PCR. The sequencing library was qualified by Qubit 2.0 (Life Technologies, USA) and Bioanalyzer 2100 (Agilent, Germany), then sequenced on Illumina Hiseq X‐Ten with 2 × 150 bp paired‐end reads, which were controlled by Hiseq Control Software (HCS).

Adaptor clipping and read trimming were done with Trimmomatic (v0.38). RNA‐seq reads were then mapped to the human reference genome (GRCh38.87) by HiSAT2 (v2.1.0). FPKM was calculated based on unique mapping reads using the StringTie package (v1.3.0). DESeq2 (v1.20.0) was used to call differentially expressed genes (DEGs). The Benjamini–Hochberg–Yekutieli procedure (implemented in the R function *p*.adjust) was used for multiple‐test correction. Significance was defined as FDR < 0.05.

### 2.4 Functional Enrichment Analysis

For assessing the biological functions of DEGs in TCM syndrome, we performed functional enrichment analysis with GeneAnalytics. Score > 10 and gene count > 5 were the cut‐off criteria.

### 2.5 qRT‐PCR

According to the manufacturer's instructions, the TRIzol reagent (Invitrogen) was used to isolate total RNA. The Prime Script RT reagent kit (TaKaRa, Dalian, China) reverse‐transcribed RNA to complementary DNA. Then, the SYBR Green master mix (TaKaRa) was used to perform a PCR with glyceraldehyde 3‐phosphate dehydrogenase and U6 as the internal control. The level of mRNAs was analyzed by the comparative 2^−ΔΔCt^ method.

### 2.6 Statistical Analysis

Principal components analysis (PCA), linear discriminant analysis (LDA), and kernel density estimation (KDE) were performed with MATLAB (R2015b). The area under the receiver operating characteristic curve (AUC) was determined with package “pROC” in R4.0.2 (http://r‐project.org). Logistic regression analysis was used to develop a diagnostic model with the package (glmt).

### 2.7 Sample Size Estimation

The sample size was calculated by Medcalc software. With a sample size ratio 1:1 in the negative and positive groups and a power of 0.9, a minimum of 22 (total sample size) was required to achieve the expected performance (AUC = 0.85). Considering the dropout rate of about 10%, the sample size of the cohort was finally set as follows: the training group (*n* = 45) with 15 H, 15 QPD, and 15 N‐QPD; the validation group (*n* = 90) with 30 H, 30 QPD, and 30 N‐QPD.

## 3 Results

### 3.1 Clinical Characterization of the Study Population

The clinical and pathological features of all subjects are presented in Table 1. A total of 135 subjects were divided into the training group (*n* = 45) with 15 H, 15 QPD, and 15 N‐QPD and the validation group (*n* = 90) with 30 H, 30 QPD, and 30 N‐QPD, which contained 50.37% (68/135) females and 49.63% (67/135) males. The average age of the two groups is 58.39 ± 11.33 and 59.37 ± 10.08, respectively. There were 47 (52.22%) early‐stage (TNM stage I and stage II) patients and 43 (47.77%) advanced‐stage (TNM stage III and stage IV) patients.

**TABLE 1:** The baseline chart of patients in this study.

Parameter	Total	Training cohort			Validation cohort		
		H	QPD	N‐QPD	H	QPD	N‐QPD
*n*	135	15	15	15	30	30	30
Age		58.39 ± 11.33			59.37 ± 10.08		
		56.24 ± 7.28	60.21 ± 9.43	59.77 ± 11.17	55.44 ± 7.08	62.90 ± 9.12	59.93 ± 10.21
Gender							
Female	68	20 (5/8/7)			48 (14/16/18)		
Male	67	25 (10/7/8)			42 (16/14/12)		
TNM stage							
I	36	—	6	8	—	12	10
II	11	—	3	2	—	3	3
III	6	—	1	1	—	2	2
IV	37	—	5	4	—	13	15

Abbreviations: H: healthy control; N‐QPD: lung adenocarcinoma patients with non‐QPD; QPD: lung adenocarcinoma patients with QPD.

### 3.2 Identification of DEGs

**FIGURE 2:**
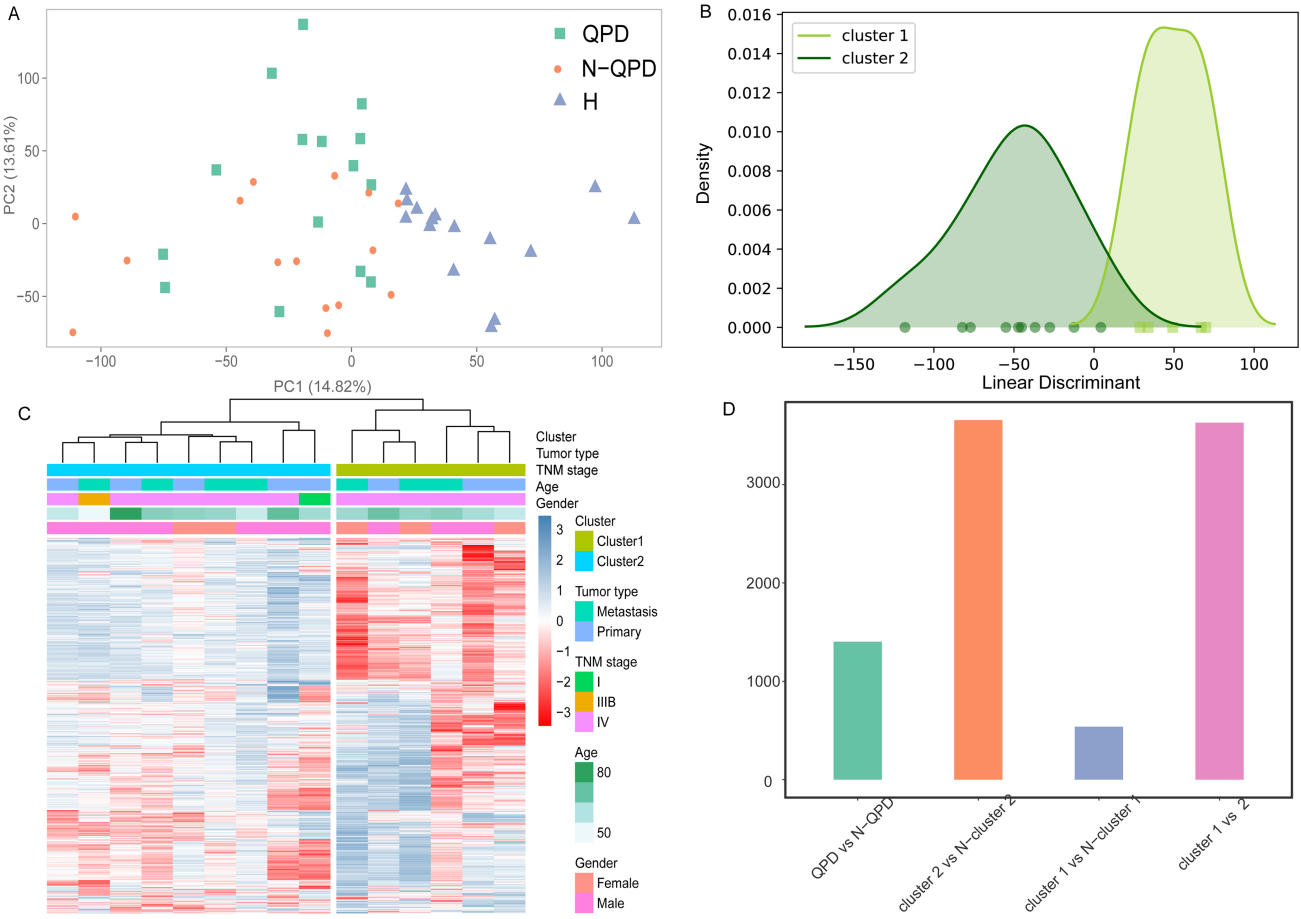
Identification of differentially expressed genes (DEGs). (A) PCA among three groups in the training cohort. (B) hierarchical clustering divided the QPD group into Cluster 1 and Cluster 2. (C) The LDA and the KDE showed multiple peaks between Cluster 1 and Cluster 2. (D) DEGs between different groups. H: healthy individuals; N‐QPD: non‐Qi deficiency and phlegm dampness; QPD: Qi deficiency and phlegm dampness lung adenocarcinoma patients.

After aligning reads to the reference genome and discarding low‐quality reads, the median value of clean reads was 9.09 million, with 89.94% of clean reads mapped to the human genome. PCA demonstrated a clear boundary between LUAD samples and normal samples, and three groups (QPD, N‐QPD, and H group) gathered in the different areas (Figure 2A). Hierarchical clustering was performed to divide the QPD group into Clusters 1 and 2 (Figure 2B). The LDA and KDE showed a separation trend between Clusters 1 and 2, and a corresponding peak was present (Figure 2C). We tried to find the reason for the differentiation of the two clusters by analyzing the correlation of clinical information (gender, age, tumor type, and TNM stage) between the two clusters. However, it is clear that there is no significant difference in clinical factors between the two clusters. We identified DEGs between different groups using “DESeq2.” The |logFC| > 2 and FDR < 0.05 were set as the cut‐off values. A total of 1480 DEGs were identified between QPD and N‐QPD groups (Figure 2D).

### 3.3 Functional Enrichment Analysis

**FIGURE 3:**
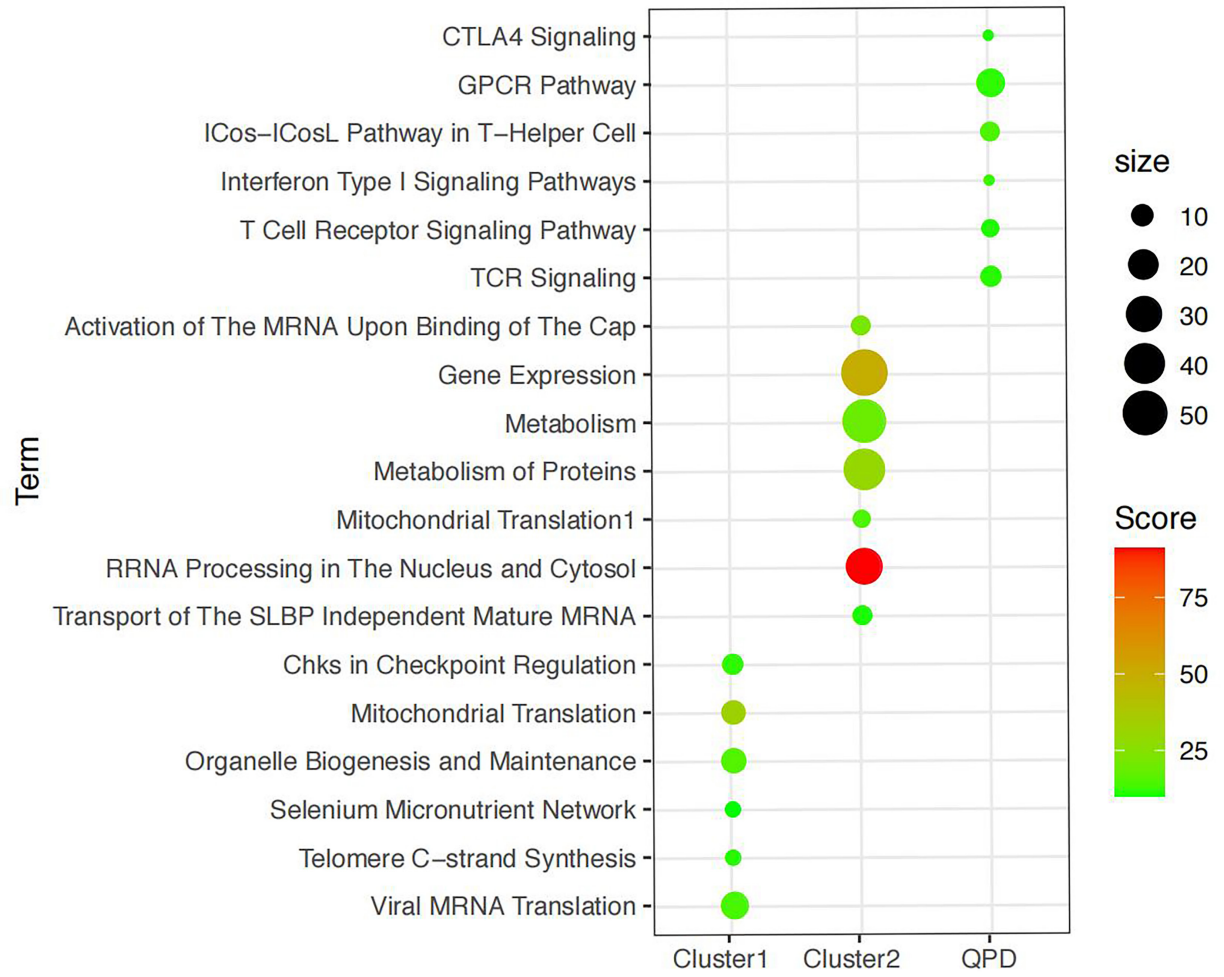
Functional enrichment analysis of DEGs.

For a better understanding of the function of DEGs, gene enrichment analysis was performed with Gene Analytics. As shown in Figure 3, biological processes were enriched in the QPD group, including CTLA4 signaling, GPCR pathway, ICos‐ICosL pathway in T‐helper cell, T cell receptor signaling pathway, TCR signaling, and T cell receptor signaling pathway. In addition, in Cluster 1, six signaling pathways were enriched, and seven were involved in Cluster 2.

### 3.4 Identification of Biomarkers for QPD Syndrome

**FIGURE 4:**
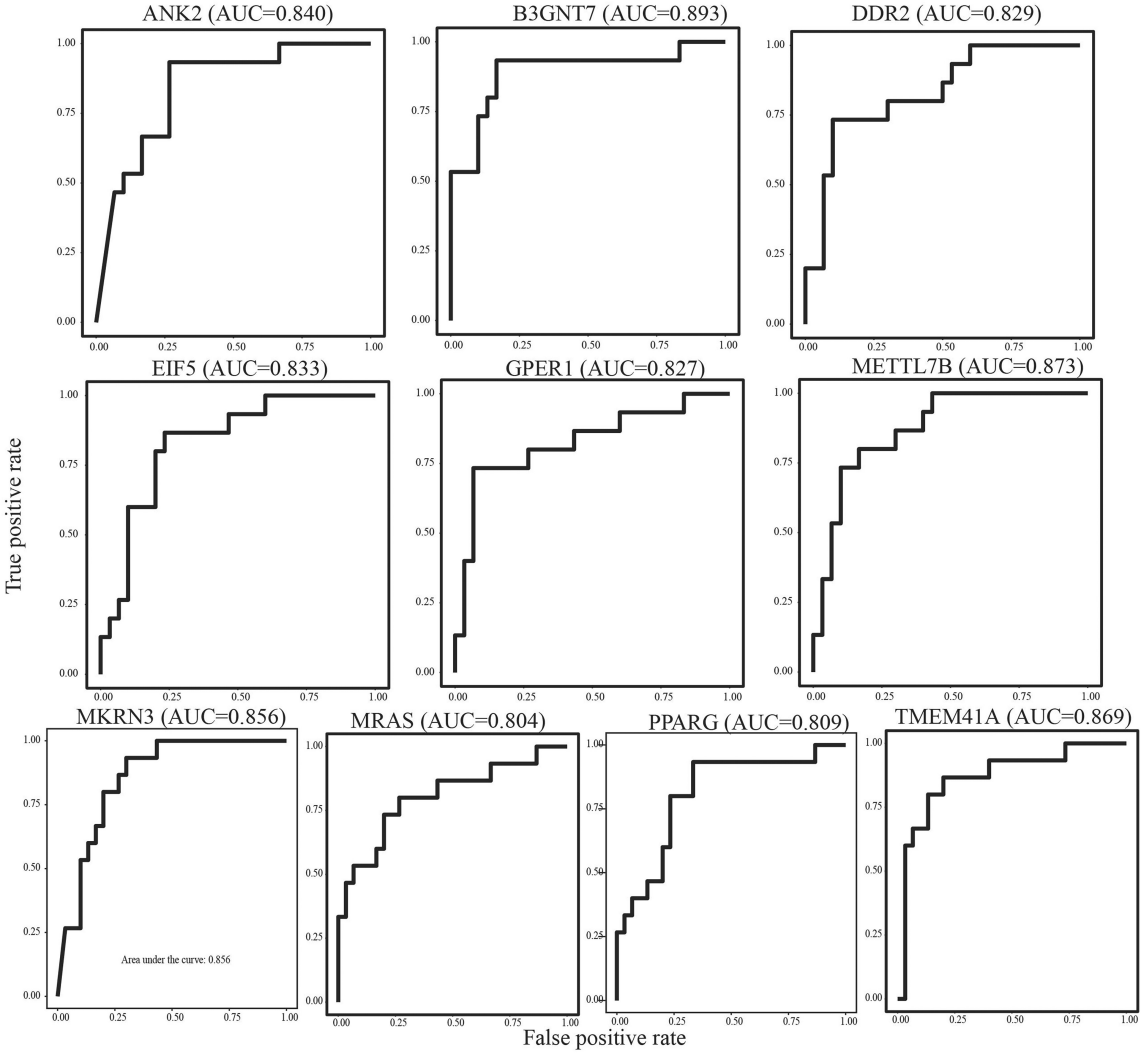
The AUC of 10 potential biomarkers (ANK2, B3GNT7, DDR2, EIF5, GPER1, METTL7B, MKRN3, MRAS, PPARG, and TMEM41A) for QPD syndrome in training cohort.

Then, the AUC of each DEG was calculated to evaluate the diagnostic effectiveness, and 10 DEGs with the highest AUC would be picked as potential biomarkers for QPD syndrome. Moreover, the DEGs highly related to selected biomarkers (*p* < 0.05) would be discarded to ensure that the biomarkers can provide as much complementary information as possible. The top 10 DEGs with AUC (ANK2, B3GNT7, DDR2, EIF5, GPER1, METTL7B, MKRN3, MRAS, PPARG (peroxisome proliferator‐activated receptor gamma), and TMEM41A) were selected as the potential biomarkers in the QPD group and presented Figure 4.

### 3.5 Validation of Biomarkers for QPD Syndrome by qRT‐PCR

**FIGURE 5:**
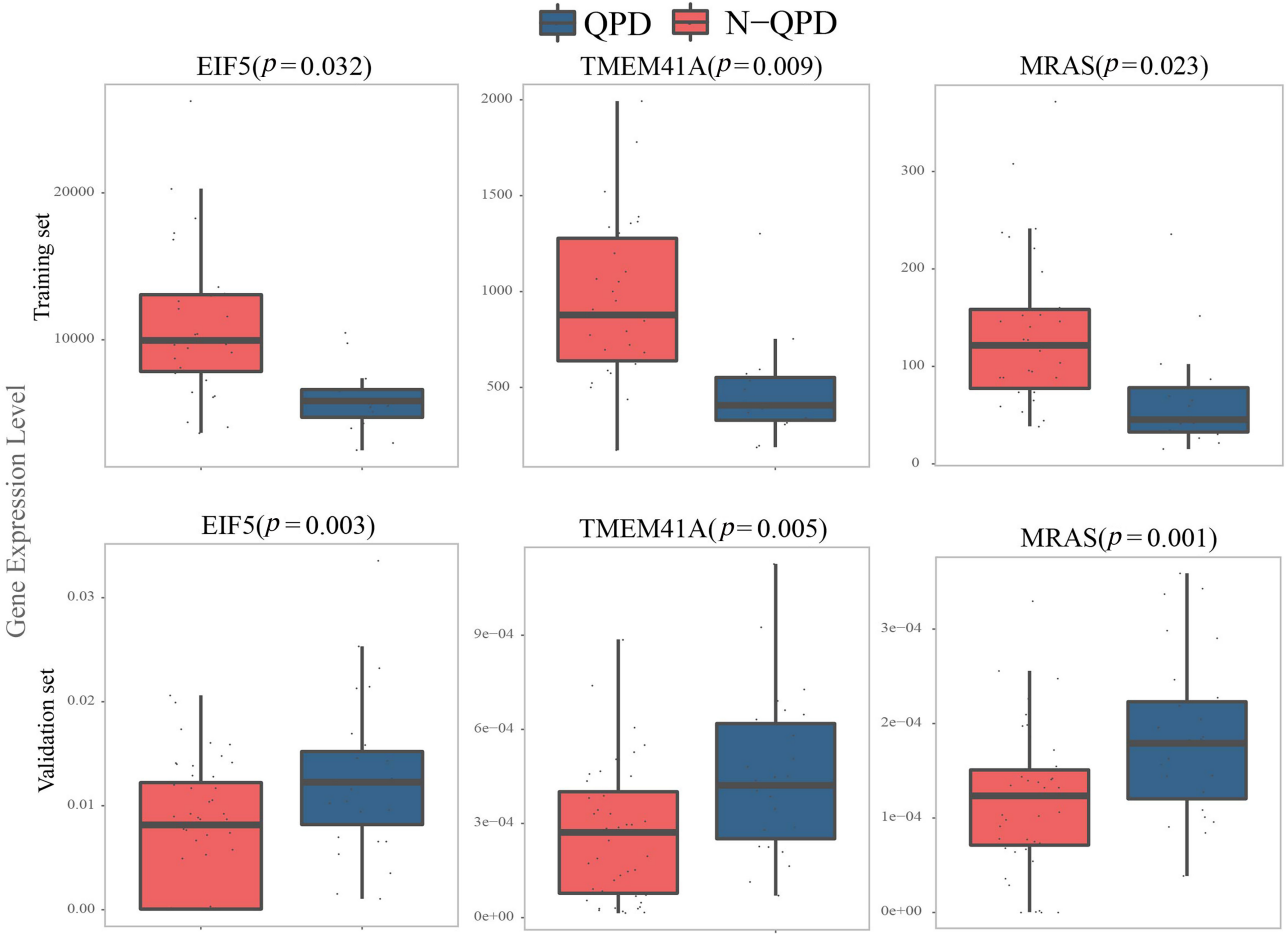
Validation of biomarkers for QPD syndrome by qRT‐PCR.

**FIGURE 6:**
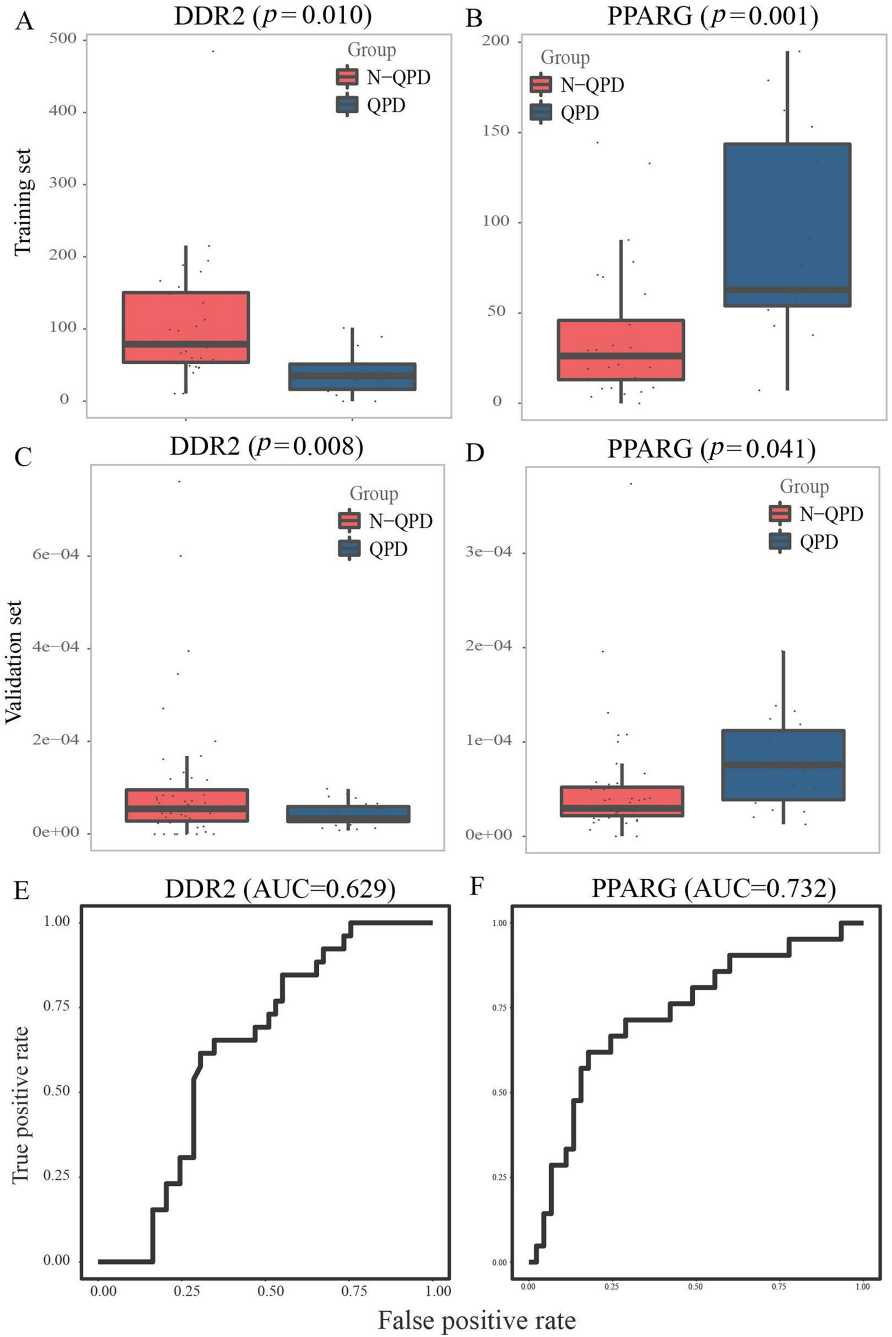
Validation of biomarkers for QPD syndrome by qRT‐PCR. (A) The expression of DDR2 in the training cohort and (C) in the validation cohort. (B) The expression of PPARG in the training cohort and (D) in the validation cohort. (E) The AUC of DDR2 for QPD syndrome in the validation cohort. (F) The AUC of PPARG for QPD syndrome in the validation cohort.

For verifying 10 biomarker expression levels in QPD syndrome, we carried out qRT‐PCR in the validation set. There was a significant difference in the expression of five genes (EIF5, TMEM41A, MRAS, DDR2, and PPARG) between the QPD and the N‐QPD groups. In addition, the results showed that the expression of three genes (EIF5, TMEM41A, and MRAS) was upregulated in the QPD group, which was inconsistent with the results of RNA‐seq (Figure 5). Besides, the expression of DDR2 was downregulated, and PPARG was upregulated, which was in line with the finding of the training set (Figure 6A–D). The AUC of DDR2 and PPARG in the validation set is shown in Figure 6E,F.

### 3.6 Development and Validation of a Diagnostic Model for QPD Syndrome

**FIGURE 7:**
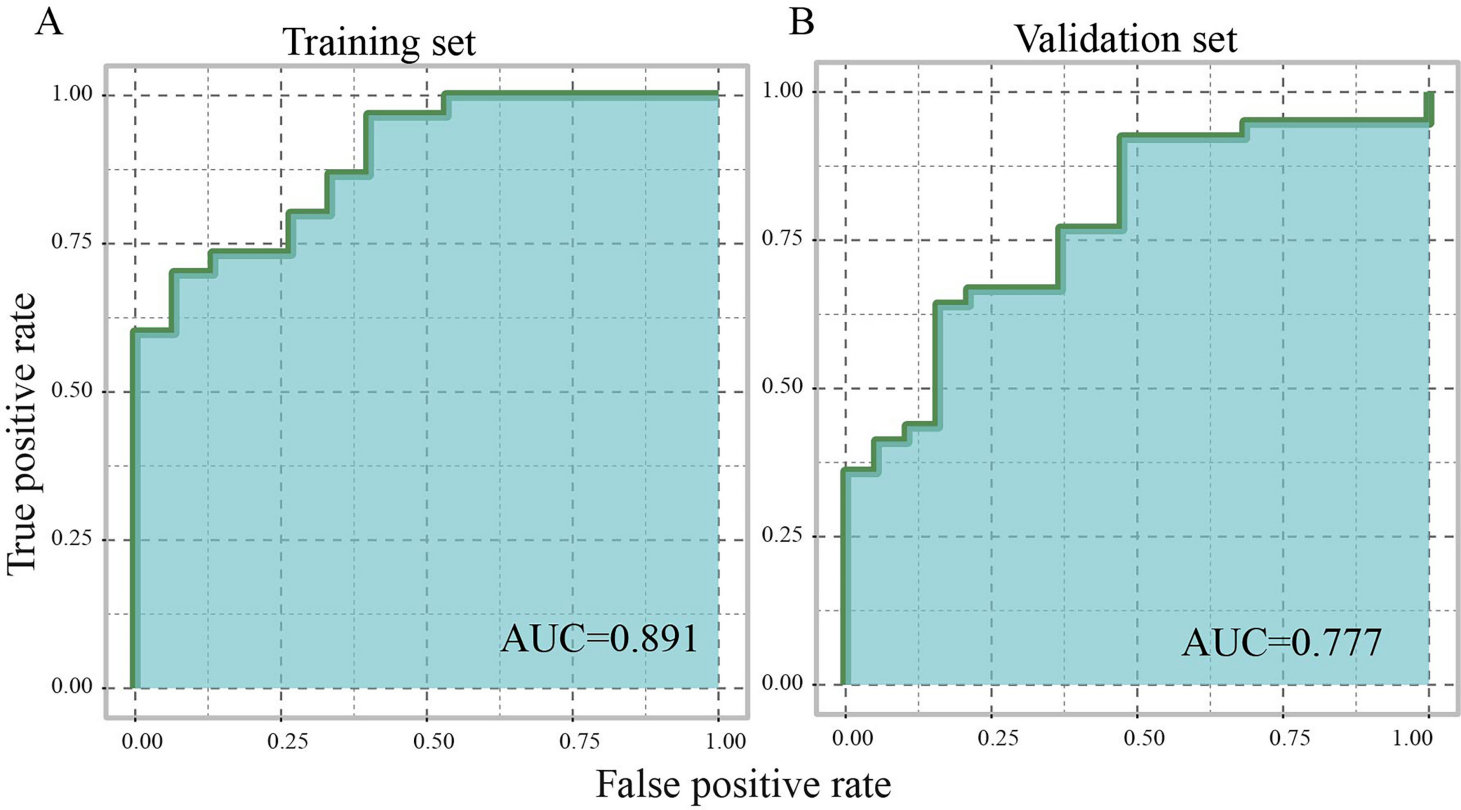
The AUCs of the diagnostic model in the training cohort and validation cohort.

Finally, we applied DDR2 and PPARG to develop a diagnostic model using logistic regression analysis. The diagnostic formula was as follows. The AUC of the diagnostic model in the training set and validation set was 0.891 and 0.777, respectively (Figure 7).

$$y=\frac{1}{1+e^{-\left(-0.4505-3.277*M\right)}}$$

$$M=\mathrm{lg}\left(\frac{\text{Expression of}\;\mathrm{DDR}2}{\text{Expression of PPARG}}\right)$$

## 4 Discussion

Comprehensive treatment or multimodal treatment has been a research hotspot in the field of cancer in recent years. In China, TCM, as a comprehensive therapy, is a common choice to improve patients' quality of life. Integrated traditional Chinese and Western medicine treatment has been reported to effectively improve some symptoms of patients with non‐small cell LC [15]. Syndrome differentiation is the core concept of TCM therapy, which is used to recognize and analyze disease syndromes. The syndrome type of patients with stage I‐III LC is mainly Qi deficiency of the lung spleen, while the syndrome type of patients with stage IV LC is mainly deficiency of both Qi and Yin. The syndrome is characterized by phlegm‐heat addiction and toxic internal obstruction, liver‐fire, and liver‐yang excess. At the same time, the deficiency is mainly caused by kidney and brain deficiency, liver‐yin deficiency, and spleen and stomach dysfunction. It is mostly caused by phlegm turbidity, blood addiction, and toxic pathogens blocking the brain collaterals and is closely related to the liver, spleen, and kidney. The clinical treatment of QPD focuses on clearing the orifices and dissipating phlegm, strengthening the body and cultivating the essence, clearing and reducing turbidity, promoting Qi, and promoting blood circulation. However, due to a lack of laboratory diagnosis and typical clinical biomarkers, the discrimination of TCM symptoms only relied on clinical symptoms. Transcriptomics is a way that applies chip technology to explore relevant genetic expression profiles, screen credible biomarkers, and provide new ideas to help explain the essence of TCM syndromes in a microscopic view [16].

In this study, we collected PBMC from LUAD with QPD syndrome and depicted gene expression profiles with RNA‐seq in the training set. We identified DEGs between groups using “DESeq2” and 1480 DEGs between QPD and N‐QPD groups. Functional enrichment analysis demonstrated that DEGs were mainly enriched in immune‐related signaling pathways. We selected top 10 highest AUC genes (ANK2, B3GNT7, DDR2, EIF5, GPER1, METTL7B, MKRN3, MRAS, PPARG, and TMEM41A) as potential biomarkers for further analysis. We validated the results in the validation set. The results showed that in QPD syndrome patients, DDR2 (discoidin domain receptor tyrosine kinase 2) was downregulated and PPARG was upregulated in both sets, which suggests that DDR2 and PPARG may be the potential biomarkers for QPD syndrome and could be used for clinical diagnosis of QPD syndrome. DDR2 encodes a member of the discoidin domain receptor subclass of the receptor tyrosine kinase protein family, which plays an important role in the communication of cells with their microenvironment [17, 18]. The activation of DDR2 was highly related to many cellular phenotypes, such as proliferation, migration, transformation, and differentiation. A previous study reported that the DDR2 mutation could influence the progression of LC by reducing the growth‐inhibitory effect of collagen [19, 20]. PPARG encodes protein, PPAR‐gamma, a member of the peroxisome proliferator‐activated receptor subfamily of nuclear receptors, and participates in the pathology of numerous diseases like obesity, diabetes, atherosclerosis, and cancer [21, 22]. It has been reported that upregulated expression of PPARG was positively associated with a better prognosis of LUAD and could drive multiple molecular triggers against the pathologic development and prognosis of LUAD [23, 24].

Molecular biology reveals the nature of biology phenomena at the molecular level, and studying the material basis of life is its main task [25]. Molecular biology has opened up a new way to understand further the inherent basic laws of life phenomena [26, 27]. Although the theories of TCM and molecular biology are the products of different thought systems and times, the material basis of life activities discussed by the two sciences is the same. Therefore, it is of great significance to introduce molecular biology into the study of TCM and study TCM from the molecular and genetic levels to promote the combination of TCM and modern medicine and realize the modernization of TCM.

In this study, we developed a diagnostic model with DDR2 and PPARG using logistic regression analysis. The AUC of the diagnostic model in the training and validation cohort was 0.891 and 0.777, respectively, suggesting that this diagnostic model was suitable for the diagnosis of QPD syndrome in LUAD.

## 5 Conclusion

We identified DDR2 and PPARG as potential biomarkers for LUAD with QPD syndrome and developed a practical diagnostic model. The result enriched the diagnostic methods of TCM syndrome differentiation, which may help to improve the accuracy and sensibility of clinical diagnosis and provide a new target for natural drug treatment of LUAD. Future studies should explore the clinical translational potential of this method in a large multicenter cohort, with a view to achieving accurate TCM diagnosis and treatment of LC earlier.

## Author Contributions

Jiabin Chen contributed to the conceptualization, reviewing, and editing of the manuscript. Sheng Wang contributed to methodology and data curation. Qiaolei Yang and Yongjun Zhang contributed to data collection and supervision. Jianfei Shen and Kequn Chai contributed to project administration. All authors have read and approved the final manuscript.

## Ethics Statement

This study was approved with informed consent by the ethics committee of Tongde Hospital, Taizhou Hospital, and Cancer Hospital.

## Conflicts of Interest

The authors declare no conflicts of interest.

## Data Availability

The data that support the findings of this study are available from the corresponding authors upon reasonable request.
